# Biomimetic Functional Nanocomplexes for Photothermal Cancer Chemoimmunotheranostics

**DOI:** 10.1002/smsc.202400324

**Published:** 2024-08-19

**Authors:** Nina Sang, Yun Qi, Shun Nishimura, Eijiro Miyako

**Affiliations:** ^1^ Graduate School of Advanced Science and Technology Japan Advanced Institute of Science and Technology 1‐1 Asahidai Nomi Ishikawa 923‐1292 Japan

**Keywords:** cancer, carbon nanohorns, chemotherapy, immunotherapy, photothermal therapy

## Abstract

This study presents a novel multimodal cancer theranostic platform developed using tumor cell‐coated biomimetic carbon nanohorn (CNH) complexes that encapsulate the anticancer drug paclitaxel (PTX). This platform combines photothermal therapy, chemotherapy, and immunotherapy to fight against malignant colorectal cancer. These engineered nanocomplexes are designed to deliver sufficient PTX molecules into a targeted solid tumor in a light‐controllable manner while inducing significant photothermal and antitumor immune responses. The outstanding photothermal conversion property of the CNHs under near‐infrared light enables effective cancer cell ablation and awakening of cytotoxic immune responses. Tumor cell membrane‐coated CNHs show improved water dispersibility, immune evasion, and targeting capabilities alongside enhanced immune activation against tumors. The efficacy of the biomimetic functional CNH nanocomplexes is demonstrated through excellent tumor‐targeting, controlled drug‐releasing behavior, and induction of cancer cell death, contributing to a robust antitumor response. This study provides a promising approach to cancer treatment by integrating multiple therapeutic modalities into a single platform, potentially enhancing treatment efficacy to combat intractable cancer.

## Graphical abstract

Cancer cell membrane (CM)‐coated biomimetic functional carbon nanohorn (CNH) complexes are developed for photothermal cancer immunotheranostics. The nanocomplexes show a high anticancer efficacy thanks to the near‐infrared‐driven synergistic physicochemical properties of CNH and the strong molecular combination of the cancer CM and chemotherapeutic drug paclitaxel.

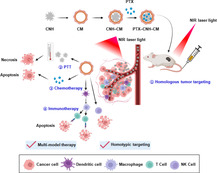

## 1 Introduction

Photothermal therapy (PTT) is a treatment strategy that uses light‐sensitive functional materials to induce irreversible cell damage through heat in targeted tissue areas. A photothermic agent acts by converting photon‐absorbed light energy into heat.^[^ ^1^ ^]^ Because temperature is a critical factor in cell dynamics and viability, PTT effectively induces hyperthermia beyond the physiological temperature of 37 °C, leading to effective inhibition of tumor mass growth.^[^ ^2^ ^]^ Thus, PTT has become one of the preferential options for designing nanotherapies for cancer research in the last decades.

Various nanomaterials have been employed and tested for PTT nanocarrier development in preclinical cancer models. In particular, inorganic nanomaterials such as gold‐, iron‐, and carbon‐based nanocomplexes are often applied for practical biomedicinal PTT developments.^[^ ^3^ ^]^ Owing to their excellent biocompatibility and potent photothermal conversion behavior, carbon nanohorns (CNHs) comprising single graphene layers forming conical and horn‐shaped tips have emerged as attractive candidates for the PTT modality.^[^ ^4^, ^5^, ^6^, ^7^, ^8^, ^9^ ^]^ In addition, their unique morphology and surface chemistry account for their efficient drug loading and targeted releasing behaviors. The conical shape of CNHs enables the effective entrapment of various drug molecules within their structure, and their surface can readily be chemically functionalized with targeting moieties such as monoclonal antibody and ligand molecules against various tumor biomarkers. Various unique physicochemical properties of CNHs make them attractive therapeutic agents for cancer treatment. However, among the various challenges, extending the systemic circulation of CNHs to maximize delivery efficiency to target sites remains a significant hurdle for future clinical nanomedical applications.^[^ ^10^ ^]^ While bare nanoparticles without functionalization or coatings often demonstrate premature leakage of loaded drugs before reaching their target sites, leading to toxic side effects, their short blood retention time further compromises their antitumor efficacy, mainly due to the immune system‐induced accelerated blood clearance.^[^ ^11^, ^12^ ^]^ Although polyethylene glycol (PEG) derivatives have been used as a nanocoating agent to improve nanoparticle biocompatibility,^[^ ^13^ ^]^ PEG still has a risk derived from toxic immune responses, such as production of anti‐immunoglobulin antibodies, which limits nanoparticle utilization in clinical trials.^[^ ^14^ ^]^ To break allowable performances of CNHs for further practical biomedical applications, simple and effective multifunctionalization of CNH with bioactive molecules is essential. Moreover, by regulating the immunogenicity of CNHs through the inclusion of more immunogenic antigens and stimulating factors, we believe that the trinity combination of immunogenic CNH with PTT and chemotherapy, which is not sufficiently explored in the field of nanocarbons, is promising as a powerful tool for future groundbreaking anticancer treatment.

Recently, modifying nanoparticle surfaces with biological cellular membranes instead of PEG coatings has been explored. This approach enables the translocation of intact membranes and peripheral proteins onto the nanoparticle surface, enhancing the delivery efficiency of nanoparticles to target sites.^[^ ^15^, ^16^ ^]^ In fact, membranes from various cells, including cancer cells, bacteria, and macrophages, were used to modify nanoparticles for anticancer vaccination, modulating antibacterial immunity and drug delivery.^[^ ^17^, ^18^, ^19^, ^20^ ^]^ In particular, cancer cell membranes (CMs) are commonly applied as single membrane coatings because they offer both immune evasion and homotypic tumor‐targeting abilities for nanoparticles.^[^ ^21^ ^]^ Self‐recognition proteins present on CMs are thought to mediate homotypic tumor‐targeting, as tumor cells strongly and mutually adhere to form primary tumors and disseminated metastases.^[^ ^22^ ^]^ Due to their unique homologous adhesion properties, CM‐wrapped nanoparticles have been studied for diagnostics, therapeutics, and anticancer vaccine development.^[^ ^23^ ^]^ The primary advantage of whole tumor cell coating is that it preserves and utilizes the functions of each cell protein, thus overcoming the limitations typically associated with ordinary nanoparticles. However, the procedures of extraction and purification of structure‐preserved CMs are generally complicated, and useful immunogenic ingredients such as cytoplasmic proteins and genes are completely removed during the processes of biomimetic nanoformulation. Moreover, further therapeutic strategies and multifunctionalization with various nanomaterials and molecules are essential for developing innovative nanomedicines that incorporate biological CMs.

**Figure 1:**
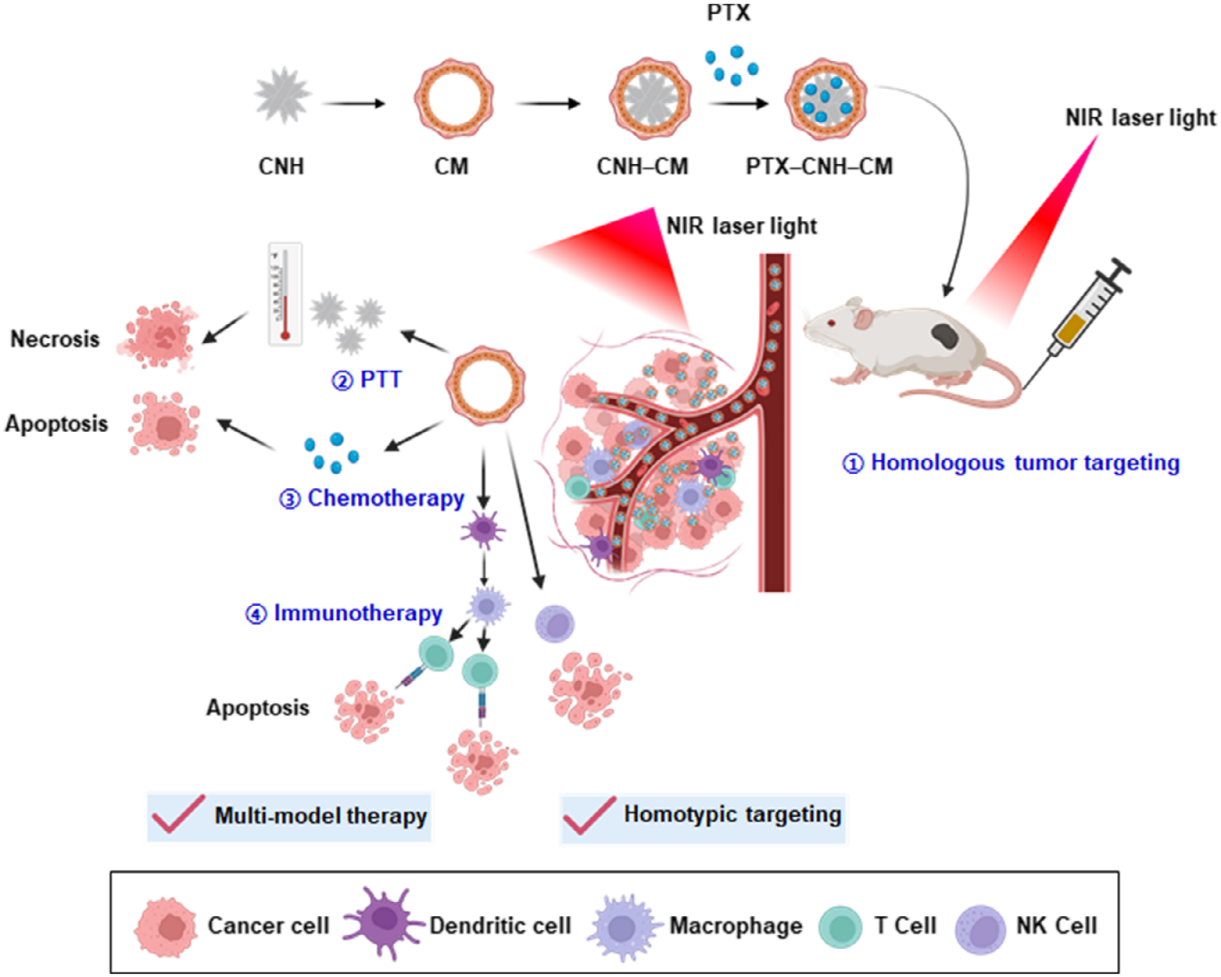
Schematic illustration of the biomimetic functional CNH complex for synergistic cancer therapy.

In this study, we first demonstrated that combining whole Colon26 cell and CNH materials could improve the immunological activity of CNHs and the delivery efficiency of the anticancer drug paclitaxel (PTX), with enhanced antitumor efficacy obtained through synergistic photothermal–chemoimmunotherapy (**Figure** **1**). We also envisioned that CM‐coated CNHs (CNH–CM) inherently mimic the surface properties of the source cells and thus acquire many unique characteristics, such as excellent water‐dispersibility, superior biocompatibility, prolonged circulation lifetimes, enhanced tumor targeting, and systemic anticancer immunogenicity.^[^ ^21^, ^22^, ^23^ ^]^ We presented a facile and effective method for synthesizing water‐dispersible CM‐coated CNH nanocomplex encapsulating PTX (PTX–CNH–CM), achieved by introducing a mouse cell line derived from Colon26 as a model CM through a simple sonication process in the presence of CNH (see Experimental Section for more details). Sonicated immunogenic Colon26 cell constructs, including cellular membranes, cytoplasmic proteins, and genes, are anticipated to act as potent immune activating antigens, improving the solubility of water‐immiscible hydrophobic PTX molecules. Our approach facilitates the preparation of PTX–CNH–CM complexes and provides them with multifunctional characteristics, including targeted drug delivery, photothermal activity, and immune activation, thereby synergistically enhancing their anticancer properties. Whole Colon26 cells that are camouflaged to bind specific tumor‐associated antigens have been wrapped around phototherapeutic CNHs to endow them with tumor‐targeting and adhesion abilities. CNHs also exhibit extraordinary photothermal qualities to eliminate tumor cells owing to their outstanding photothermal conversion efficiency, especially when irradiated with biologically permeable near‐infrared (NIR) light.^[^ ^4^, ^5^, ^6^, ^7^, ^8^, ^9^ ^]^ These multifunctional strategies for biomimetic functional CNH complexes could pave the way for new therapeutic applications of CNH materials in future clinical trials. We also believe that these unique properties of biomimetically functionalized CNH with attractive advantages could be a breakthrough for surface chemistry and further capability of CNH in various biological fields beyond cancer therapy.

## 2 Results and Discussion

### 2.1 Structural and Optical Characterization of CNH Complexes

**Figure 2:**
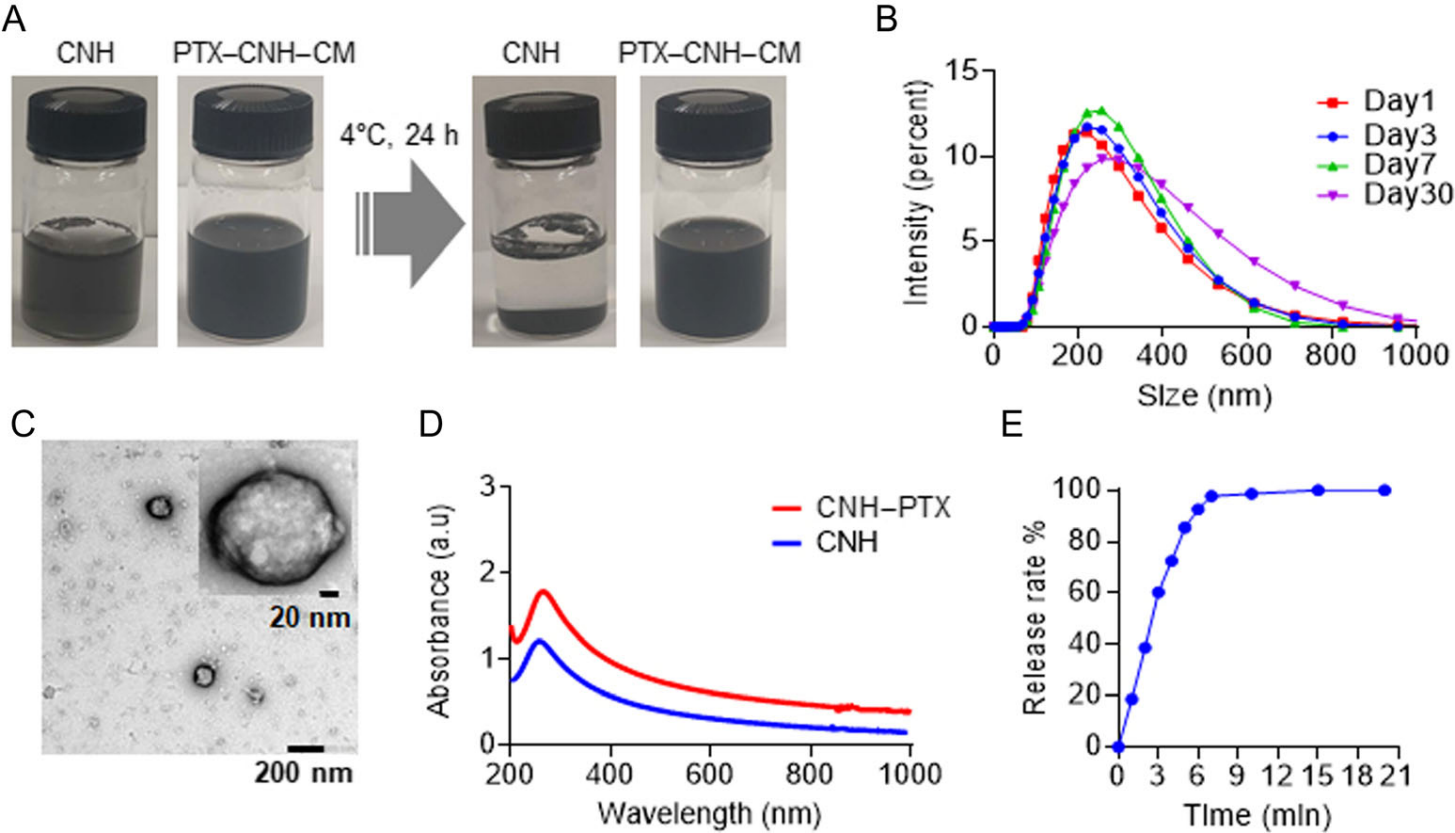
Structural and optical characterization of PTX–CNH–CM complexes. A) Images of CNH and PTX–CNH–CM in PBS buffer before and after incubation for 24 h at 4 °C. B) DLS size distribution of PTX–CNH–CM. Average hydrodynamic diameters of the PTX–CNH–CM on days 1, 3, 7, and 10 after preparation. C) TEM images of PTX–CNH–CM; the upper right image indicates a high‐magnification view of the nanocomplex. D) UV–vis–NIR absorbance spectra of PBS suspension of CNH and PTX–CNH–CM (PTX concentration = 5 μg mL^−1^ and CNH concentration = 50 μg mL^−1^). E) Drug release profile from laser‐induced PTX–CNH–CM. Data are presented as means ± standard error of the mean (SEM) (*n* = 3; independent tests).

The prepared PTX–CNH–CM exhibited a dark color and high water dispersibility (**Figure** **2**A). Dynamic light scattering (DLS) measurements revealed that the nanocomplex maintained its nanoscale hydrodynamic diameter for at least 30 days (Figure 2B), indicating excellent colloidal aqueous stability. Besides, DLS said that CNH–CM has also high water dispersibility, with an almost similar hydrodynamic diameter (≈ 219 nm) to PTX–CNH–CM (≈ 218 nm) (Figure S1A, Supporting Information). Pristine CNH without any functionalization indicated polydispersity and bigger aggregations in the DLS chart due to its low water dispersibility derived from strong hydrophobic nature of CNH (Figure S1A, Supporting Information). The zeta potentials of CNH, CNH–CM, and PTX–CNH–CM in PBS buffer were −24.7, −11.7, and −11.9 mV, respectively (Figure S1B, Supporting Information). The negative potential of CNH might be explained by the carboxyl and hydroxyl groups of CNH formed by production processes.^[^ ^24^, ^25^ ^]^ In any case, we believe that multivalent molecular interactions of cellular ingredients with CNH could influence on the zeta potential of CNH although both of PTX–CNH–CM and CNH–CM possess still negative charges. Transmission electron microscopy (TEM) analysis of PTX–CNH–CM further corroborated the spherical and coating morphologies of the individually distributed nanocomplexes, with a diameter of ≈200 nm (Figure 2C). Meanwhile, CNH–CM also displayed monodispersity on a TEM grid and a similar coating structure derived from cellular ingredients to PTX–CNH–CM (Figure S2, Supporting Information). The pristine CNH did not show any coating layers on its surface at all; it exhibited characteristic single‐graphene tubules with bare horn‐shaped tips and massive aggregations due to the aforementioned poor water‐dispersibility (Figure S2, Supporting Information). Besides, thermogravimetric analysis (TGA) showed that ≈0.74 mg of CM and 0.09 mg of PTX were coated on surface of 1 mg of CNH (Figure S3, Supporting Information). The ultraviolet–visible–near‐infrared (UV–vis–NIR) optical absorption spectra of the CNH–CM and PTX–CNH–CM dispersions in the aqueous solution were determined (Figure 2D). CNH–CM exhibited appreciable absorbance in the NIR region (700–1000 nm) from CNH, with a peak around 200–300 nm attributed to the PTX and CNH components.

CNHs outperform inorganic nanomaterials, such as metric nanoparticles and nanorods, due to their high drug loading capacity facilitated by their large surface area via CNH–drug substrate hydrophobic interactions.^[^ ^26^ ^]^ We are sure that a cellular membrane coating onto drug‐loaded CNHs can prevent premature drug leakage while improving circulation and tumor delivery properties. The drug loading efficiency of the PTX–CNH–CM nanocomplex was evaluated by calculating unbound PTX molecules after filtration using a UV–vis–NIR spectrometer. Eventually, ≈86.4% of the PTX was successfully loaded onto the nanocomplex. More interestingly, using an 808 nm NIR laser at 1.2 W (≈ 61.1 mW mm^−2^) could spatiotemporally release PTX molecules (Figure 2E). A fiber‐coupled 808 nm continuous‐wave diode laser was employed in this study due to its high biopermeability, convenient visibility for targeting objectives, and inexpensive commercial availability. Overall, these results indicate that the CNH–CM complex can work as a light‐controllable drug carrier for cancer treatment.

**Figure 3:**
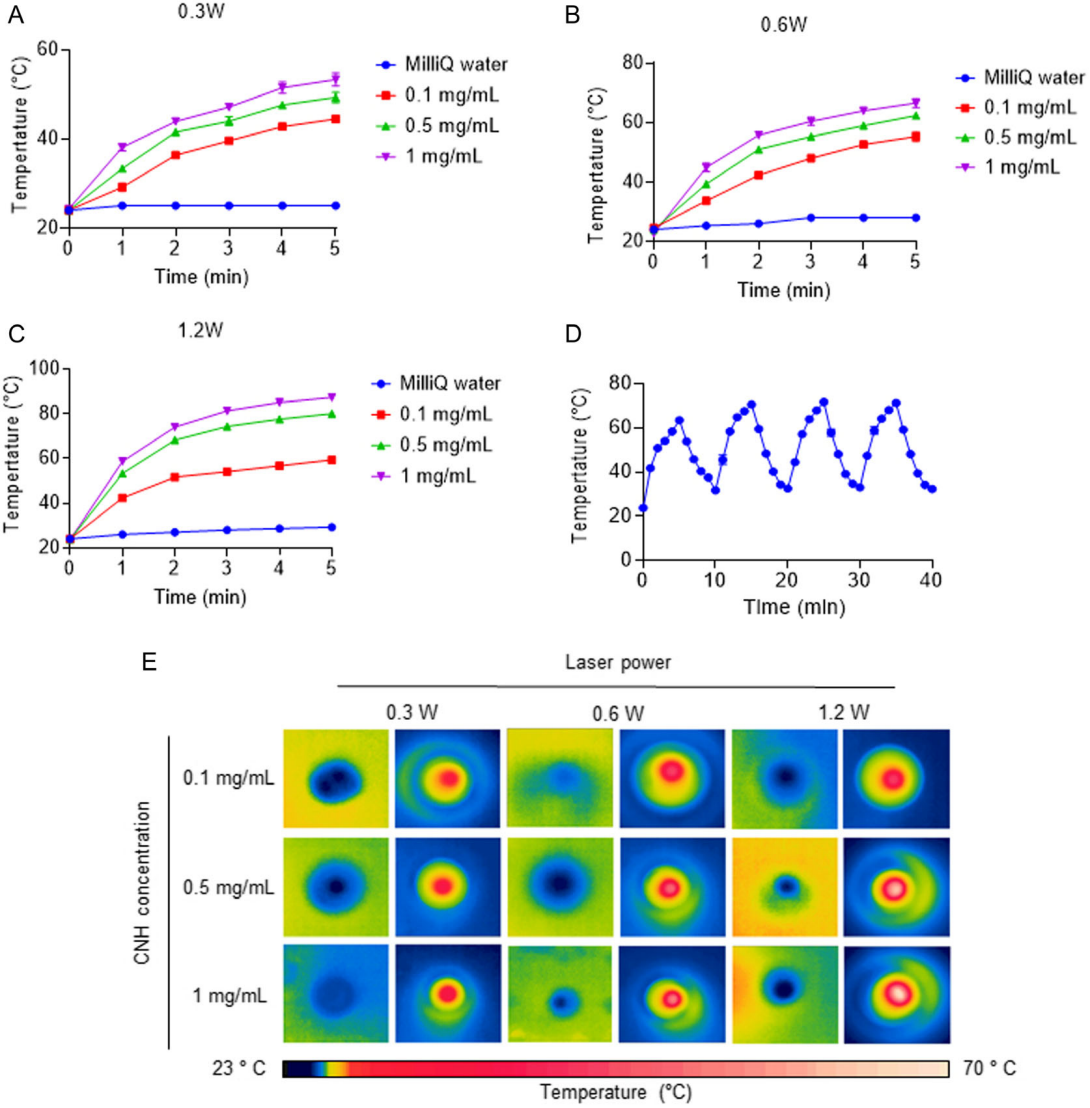
Photothermal conversion properties of the CNH complex. A–C) Laser‐induced temperature increase in MilliQ water (control) and PTX–CNH–CM suspensions at different CNH concentrations by 808 nm laser irradiation at 0.3 W (15.3 mW mm^−2^), 0.6 W (30.6 mW mm^−2^), or 1.2 (60.12 mW mm^−2^) power. CNH (1 mg mL^−1^) was dispersed in 0.2 mL of PBS. Data are presented as means ± SEM (*n* = 3; independent tests). D) Photothermal stability test of the PTX–CNH–CM solution under photothermal heating and natural cooling cycles by 808 nm laser irradiation at 1.2 W (≈61.1 mW mm^−2^) power. CNH (1 mg mL^−1^) was dispersed in 0.2 mL of PBS. Data are presented as means ± SEM (*n* = 3; independent tests). E) Thermographic images of PTX–CNH–CM solution after 5 min laser irradiation at various laser powers 0.3 W (≈15.3 mW mm^−2^), 0.6 W (≈30.6 mW mm^−2^), and 1.2 W (≈61.1 mW mm^−2^) and different CNH concentrations (0.1, 0.5, and 1.0 mg mL^−1^).

Next, the photothermal conversion ability of the PTX–CNH–CM complex was evaluated using a thermocouple under 808 nm laser irradiation. Although the depth of tissue penetration is somewhat limited in the NIR light itself in comparison with X‐ray and ultrasound, utilizing light in the NIR region offers the potential to effectively visualize and treat solid tumors with enhanced precision and heating efficiency by using a highly focused laser beam with powerful photothermal nanomaterials. We believe that the use of NIR light promises to improve cancer phototherapy by enabling the selective delivery of increased therapeutic energy to tissues at substantially greater depths. We choose an 808 nm laser as a model light source for the current study because of the excellent visibility of a laser beam for determine a targeting cite and the low absorption coefficient for water molecules and other ingredients in biological tissues to avoid excess heating for animal experiments. The temperature of PTX–CNH–CM suspension (CNH concentration = 1 mg mL^−1^) was rapidly reached around 58 °C under 0.3 W (15.3 mW mm^−2^) laser power density (**Figure** **3**A). This substantial temperature rise certainly results from the photothermal conversion ability of the PTX–CNH–CM complex that can effectively convert laser light energy into thermal energy. Temperature increases were easily contrivable by using different laser power densities of 0.6 W (30.6 mW mm^−2^) and 1.2 W (61.1 mW mm^−2^) at different PTX–CNH–CM concentrations (Figure 3B,C). Such dose‐dependent thermal responses highlight the scalability and adaptability of the PTX–CNH–CM complex to varying therapeutic requirements. Given the paramount importance of stability in ensuring consistent treatment efficacy and minimizing potential thermal damage to surrounding healthy tissues during PTT, we measured the temperature changes (ΔT) in PTX–CNH–CM suspension during laser on/off cycles (four cycles of deactivation and activation) (Figure 3D). This analysis revealed negligible fluctuations and a marginal decline in temperature, thereby demonstrating the satisfactory photothermal stability of the studied complex. This result supports the robustness of PTX–CNH–CM complexes under thermal stress and affirms their reliability as agents in precision‐targeted cancer PTT. We also confirmed the powerful photothermal conversion property of the studied complex by thermographic images of PTX–CNH–CM solutions at different concentrations before and after irradiation with a fiber‐coupled 808 nm continuous‐wave NIR laser for 5 min (Figure 3E). The photothermal conversion efficiency of PTX–CNH–CM was 63%. The efficiency of PTX–CNH–CM was greater than those of other photothermal nanomaterials, such as metal‐based materials, carbon dots, and semiconductor polymer nanoparticles (Table S1, Supporting Information).^[^ ^27^, ^28^, ^29^ ^]^

### 2.2 In Vitro Anticancer Efficacy of the CNH Complex

**Figure 4:**
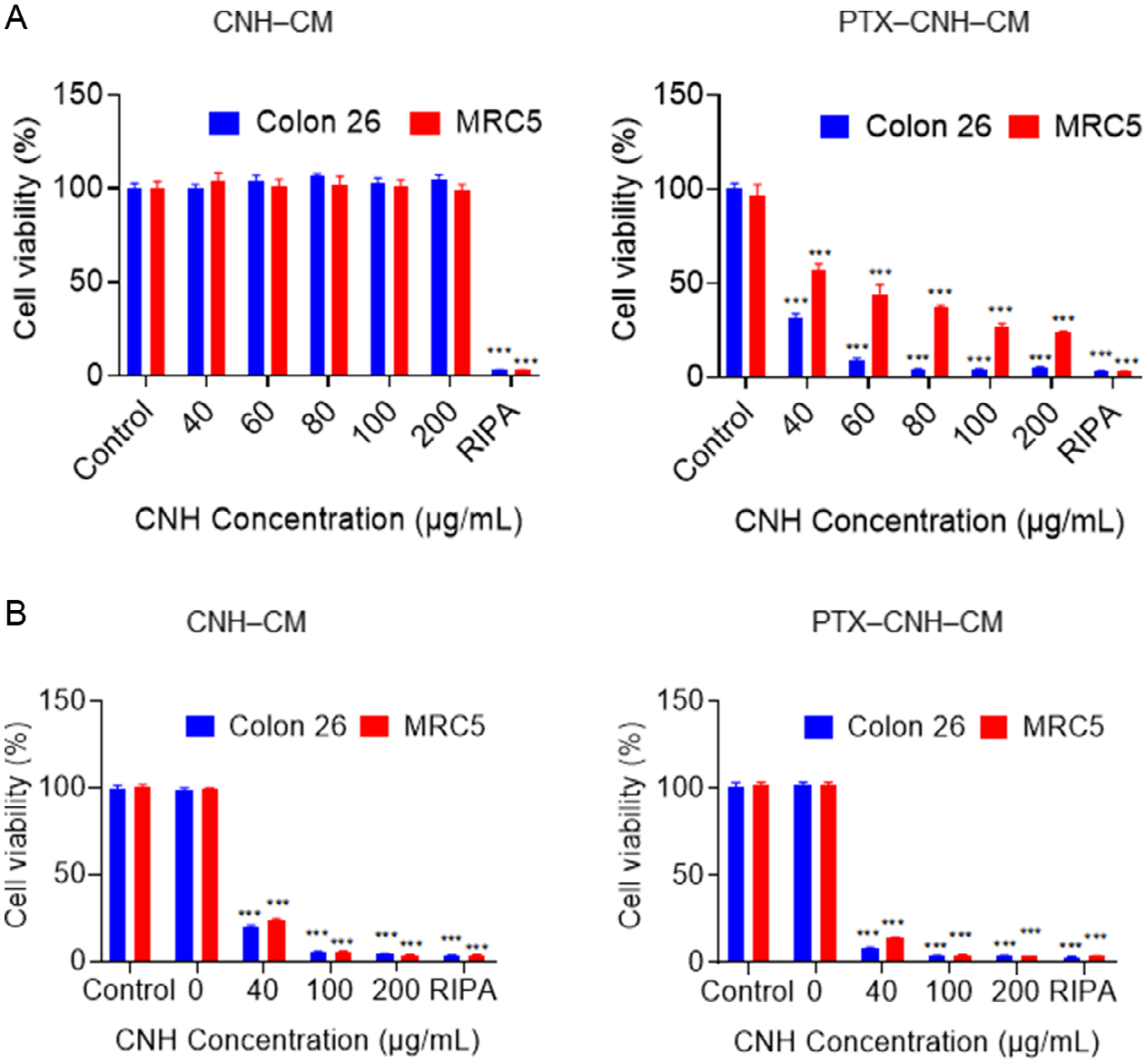
Laser‐induced cytotoxicity of the CNH complex. A) Viability of Colon26 and MRC5 cells treated with the CNH**–**CM, PTX**–**CNH**–**CM, and RIPA buffer (control) at various CNH concentrations. Cell viability was tested 24 h after treatment. Data presented as means ± SEM (*n* = 5; biologically independent tests), ***p < 0.001 versus control without nanoparticles (Student's *t*‐test). B) Laser‐induced cytotoxicity evaluation in Colon26 and MRC5 cells exposed to the CNH**–**CM, PTX**–**CNH**–**CM, and RIPA buffer (control) after 24 h of treatment with 5 min laser irradiation (0.3 W [≈15.3 mW mm^−2^]) at various CNH concentrations. Data presented as means ± SEM (*n* = 5; biologically independent tests), ***p < 0.001, by Student's *t*‐test.

Subsequently, the in vitro anticancer efficacy of the laser‐activated PTX–CNH–CM complex against the human normal diploid fibroblast cell line (MRC5) and Colon26 cells was assessed (**Figure** **4**). Before exploring the photothermic and chemotherapeutic anticancer efficacy, the potential cytotoxicity of CNH–CM was evaluated by incubating the cells with varying concentrations of CNH–CM for 24 h without NIR laser exposure (Figure 4A). Notably, at all tested concentrations, these CNH–CMs alone showed no cytotoxic effects on either the MRC‐5 or Colon26 cells. In contrast, the radioimmunoprecipitation (RIPA) lysis buffer, serving as a positive control, displayed severe cytotoxicity toward both cell types. Meanwhile, the PTX–CNH–CM complex induced significant cytotoxic effects on both Colon26 and MRC5 cells thanks to the anticancer agent PTX. Notably, the PTX–CNH–CM complex showed higher cytotoxicity toward Colon26 cells than toward normal MRC5 cells, indicating the heightened sensitivity of cancer cells to PTX due to selective biochemical responses of drug molecules.^[^ ^30^ ^]^ The cytotoxic effects of laser‐activated CNH–CM and PTX–CNH–CM complexes were then investigated (Figure 4B). Under 808 nm NIR laser irradiation at 0.3 W (≈61.1 mW mm^−2^), CNH–CM effectively eradicated Colon26 and MRC5 cells, leveraging the robust photothermal conversion capability of CNH. Moreover, the laser‐activated PTX–CNH–CM complex enhanced cytotoxicity toward both Colon26 and MRC5 cells, attributed to the synergistic impact of PTX release and the powerful photothermal conversion of CNH.

**Figure 5:**
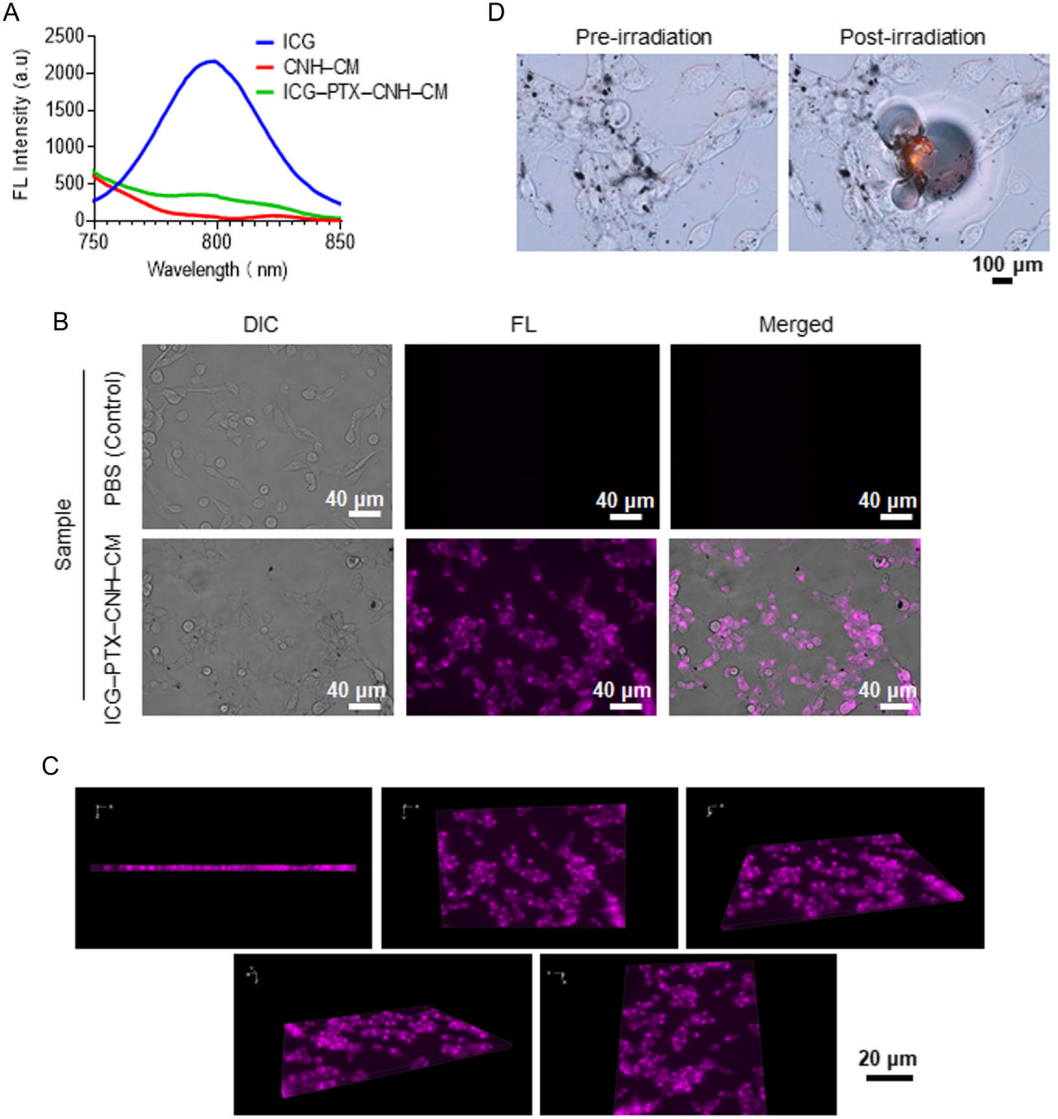
Intracellular distribution of the CNH complex. A) FL spectra of ICG (ICG concentration = 12.5 μg mL^−1^), CNH–CM (CNH concentration = 125 μg mL^−1^), and ICG–PTX–CNH–CM (CNH concentration = 125 μg mL^−1^, ICG concentration = 12.5 μg mL^−1^, and ICG–PTX concentration = 31.3 μg mL^−1^) at 750 nm excitation wavelength. B) FL bioimaging of Colon26 cancer cells after incubation with PBS (control) and ICG–PTX–CNH–CM. The 2D pictures represent differential interference contrast (DIC), FL, and a merged image (DIC + FL). Pink and black particles represent ICG–PTX–CNH–CM. C) 3D FL images of Colon26 cells after incubation with ICG–PTX–CNH–CM. D) Real‐time observation of Colon26 cancer cell destruction by laser‐induced PTX–CNH–CM before and after laser irradiation (808 nm, 564 mW, ≈287 mW mm^−2^). The red circle represents the laser irradiation position and area.

To evaluate the targeting ability of the CNH complex, the uptake and interaction of CNH complexes and Colon26 cells were examined via fluorescence (FL) spectrometer and FL microscopy. To this end, the PTX–CNH–CM encapsulating NIR fluorescent molecule indocyanine green (ICG) (ICG–PTX–CNH–CM) was synthesized through a simple sonication process (refer to the Experimental Section). The PBS‐insoluble ICG, alongside PTX, is sequestered within the lipid‐mediated hydrophobic region of the CM. The FL spectrometer revealed no NIR FL emission from ICG upon NIR light excitation in the synthesized ICG–PTX–CNH–CM, presumably owing to CNH–ICG molecular interactions (**Figure** **5**A).^[^ ^31^, ^32^ ^]^ ≈99.6% of the ICG was loaded onto the nanocomplex by confirming with the same way as calculation of PTX loading efficacy (Figure S4, Supporting Information). About 87.7% of ICG was disattached from CNH after filtration with DMSO, resulting in restoring FL property of ICG in filtrates (Figure S4, Supporting Information). These results clearly indicate that ICG molecules were surely conjugated with CNH–CM. After a 24 h incubation of ICG–PTX–CNH–CM with Colon26 cells at 37 °C, intracellular uptake of ICG–PTX–CNH–CM (as black and/or pink dots) was observed in cells, indicating the detectability of ICG postrelease from the nanocomplexes (Figure 5B). In contrast, the control cells showed no such FL, differing from the ICG–PTX–CNH–CM complex outcomes. 3D FL microscopy further demonstrated uniform subcellular distribution of ICG–PTX–CNH–CM within the cytosol (Figure 5C).

The real‐time anticancer activity of the NIR laser‐induced ICG–PTX–CNH–CM complex was assessed using a single laser beam integrated with the FL microscopy setup. Remarkably, Colon26 cell structures were instantaneously destructed, forming bubbles presumably from water vaporization caused by the intense photoexothermic reaction of CNH, upon 808 nm laser irradiation at 564 mW (≈287 mW mm^−2^) (Figure 5D; Supporting video 1, Supporting Information). Control experiments without ICG–PTX–CNH–CM complexes did not show any cancer cell or spheroid destruction (see Supporting video 2, Supporting Information). These findings demonstrate the potent photothermal conversion capacity of CNH complexes in precisely targeting and eradicating cancer cells.

### 2.3 In Vivo Targeting and Anticancer Effect of CNH Complexes

**Figure 6:**
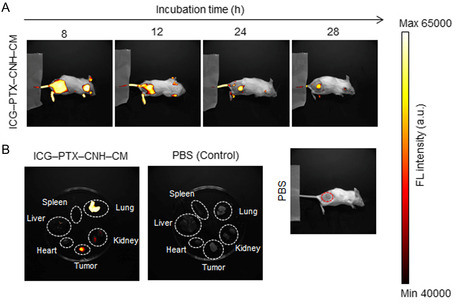
Systemic distribution of the CNH complex in the tumor model. A) FL imaging of Colon26 tumor‐bearing mice after i.v. injection of PBS and ICG–PTX–CNH–CM. B) Extracted vital organs and tumors after an i.v. injection of PBS or ICG–PTX–CNH–CM (ICG, 11.11 mg kg^−1^; and CNH, 5.6 g kg^−1^) (200 μL, ICG, 1 mg mL^−1^; and CNH, 1 mg mL^−1^). The red dashed circle denotes the solid tumor location.

The systemic distribution of functional tumor cell‐modified CNH complexes was investigated to clarify the tumor‐targeting and anticancer eradication ability of the CNH complex. We used NIR fluorescent ICG–PTX–CNH–CM in a Colon26‐bearing syngeneic mouse model using an in vivo bioimaging system. The tumor‐targeting distributions of the CNH complexes were observed over time in tumors after intravenous (i.v.) injections of ICG–PTX–CNH–CM through the tail vein of mice, owing to the enhanced permeability and retention (EPR) effects (**Figure** **6**A).^[^ ^33^ ^]^ The control (PBS injection) showed no FL throughout the entire body of the injected mouse. Although slight FL was observed in the extracted lungs, the targeted tumor exhibited a stronger FL intensity (Figure 6B). The FL observed in the lungs and kidneys may result from the excellent water dispersibility of the CNH complexes, which enhanced their long‐term blood circulation, as the major elimination pathway for the CNH complexes is renal excretion. More interestingly, ICG–PTX–CNH–CM exhibited higher tumor targeting rather than the conventional CNH solution with a dispersant Cremophor EL and NIR fluorescent dye ICG (ICG–CNH–CRE) because of the advantages of CM such as high water‐dispersibility, excellent biocompatibility, prolonged circulation lifetimes, and enhanced tumor penetration ability (Figure S5, Supporting Information). Collectively, these results indicate that the prepared ICG–PTX–CNH–CM have high tumor selectivity and longer tumor site retention for a single administration. As the success of any therapy hinges on its ability to reach most of the tumor volume, these results suggest that designing a tumor‐selective CNH complex is an innovative way to target and anchor to a larger cell subpopulation deep within and throughout the tumor stroma.

**Figure 7:**
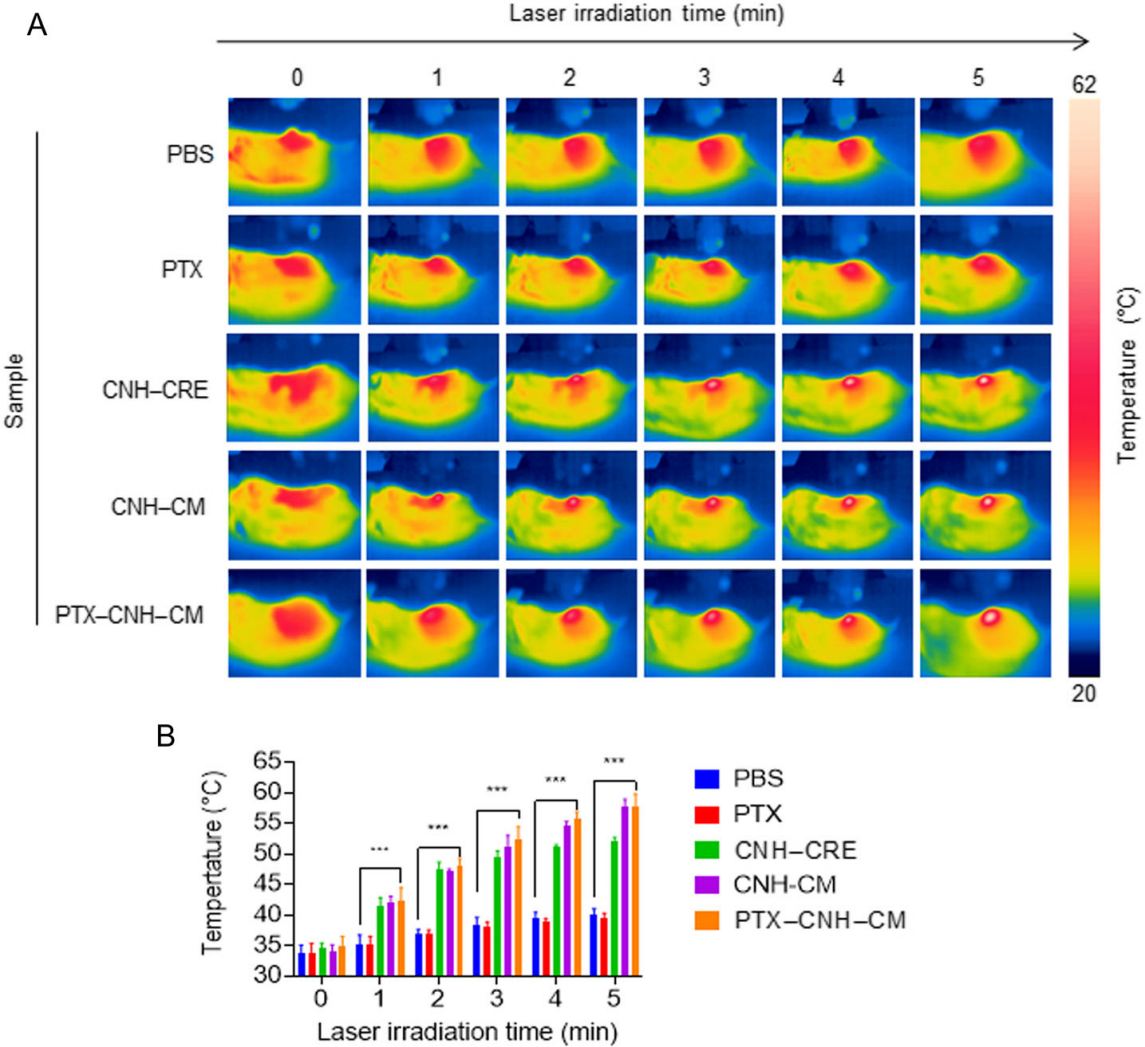
In vivo photothermal conversion behavior of laser‐induced PTX–CNH–CM. A) Thermographic measurement of the tumor surface by treatment with PBS or PTX–CNH–CM‐i.v. injected mice. Laser power, irradiation time, and wavelength are 0.6 W (≈30.6 mW mm^−2^), 5 min, and 808 nm. B) Temperature changes of tumors in Colon26‐bearing mice on day 2 after injection with PTX–CNH–CM or PBS followed by 808 nm laser irradiation for 5 min [laser power = 0.6 W (≈30.6 mW mm^−2^)]. Data are expressed as means ± SEM; *n* = 5 independent experiments. Statistical significance was calculated in comparison with the PBS group. ***p < 0.001, by Student's *t*‐test.

After the CNH complex precisely reaches the tumor tissue, we continue to test the photothermal ability of the nanocomplex. The targeted Colon26 tumor of a mouse was then irradiated using an 808 nm laser at 600 mW (≈30.6 mW mm^2^) after 24 h of a single i.v. administration of each sample. The body surface temperature was continuously monitored using a thermographic camera during irradiation (**Figure** **7**A). The tumor surface temperatures of mice injected with tumor CM‐coated PTX–CNH–CM markedly increased after 5 min of NIR irradiation, reaching a maximum value of ≈58 °C (Figure 7B). Although the CNH solution with a dispersant Cremophor EL (CNH–CRE) also showed marked temperature increases, the laser‐induced PTX–CNH–CM and CNH–CM demonstrated higher tumor surface temperature growth than laser‐induced CNH, indicating that PTX–CNH–CM and CNH–CM accumulated in solid tumors and effectively heated the deeper internal tumor tissue due to the EPR effect, binding of PTX–CNH–CM onto the proteins of the Colon26 tumor cells, and phagocytosis by myeloid cells, such as macrophage and neutrophil owing to the immunological activation by the CMs (Figure 7B). In contrast, the control groups (PBS and PTX) displayed slight surface temperature increases after laser irradiation, probably because the skin, blood, and tissue converted light energy to heat. This exhaustive evaluation not only corroborates the precise tumor‐targeting and thermal induction properties of CNH–CM complexes but also accentuates their significant promise as an impactful instrument in the fight against cancer. It underscores the strategic advantage of combining targeted delivery with the synergistic benefits of PTT, illustrating the nanoparticles’ capacity to navigate and disrupt cancerous tissues effectively.

**Figure 8:**
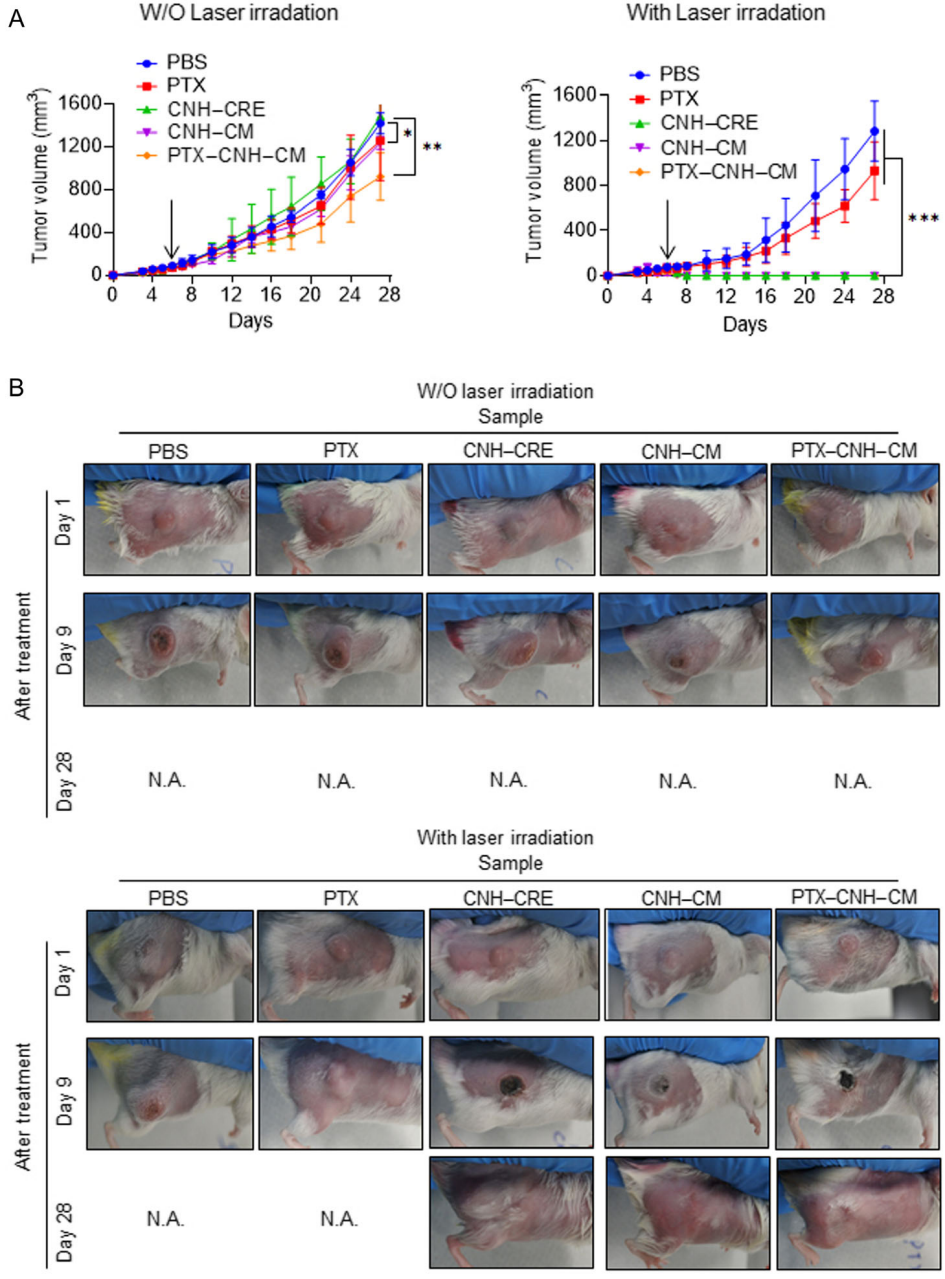
In vivo anticancer test of light‐induced CNH complexes. A) Anticancer efficacy of various samples with and without laser irradiation. PBS or dispersion of PTX, CNH, CNH–CM, and PTX–CNH–CM was intravenously injected into the Colon26‐bearing mice. After 24 h, the tumors were treated with 808 nm laser irradiation (laser power = 0.6 W (≈30.6 mW mm^−2^); irradiation time = 5 min every day (total 2 times irradiation). Data are expressed as means ± SEM (*n* ≥ 4 biologically independent tests). *p < 0.05, ***p < 0.001, by Student's *t*‐test. The black arrows display the time point of sample administration. B) Photos of the mice after each treatment.

The CNH–CRE complexes did not exhibit any therapeutic effect against the tumor without laser irradiation due to their lack of photothermal conversion properties (**Figure** **8**A,B). In contrast, PTX alone displayed better efficacy than the PBS group, while the CNH–CM showed nearly the same suppression of tumor growth as PTX, which might be attributed to immune activation and the targeting effect of the nanoparticles. The PTX–CNH–CM demonstrated better inhibition of tumor growth than either the PTX or CNH–CM group at the same amount of PTX or CNH–CM. This superior performance of PTX–CNH–CM could be due to the synergistic effect of both PTX and the immune responses elicited by the cancer CM.

Laser‐induced anticancer therapeutic efficiency was further investigated using a Colon26‐bearing model. The PTX–CNH–CM + laser group exhibited the highest anticancer efficacy compared to the other control groups (Figure 8A,B). In fact, the irradiated solid tumors completely disappeared after just two rounds of laser irradiation with CNH–CRE, CNH–CM, and PTX–CNH–CM. This was attributed to the excellent photothermal property of CNH, the exceptional tumor‐targeting effect, the effective controlled drug‐releasing of PTX, and the multidimensional immunological stimulations, resulting in a 100% complete response (CR) rate at the 28 day follow‐up. The CNH–CRE + laser group also indicated CR of the tumor due to the potent photothermal conversion. The PTX + laser group showed somewhat stronger antitumor effectiveness than the PBS + laser group, likely due to the antitumor effect of PTX. These results indicate that laser induction might help in immunological activation by the heat energy from the natural photothermal conversion of biological tissue.

**Figure 9:**
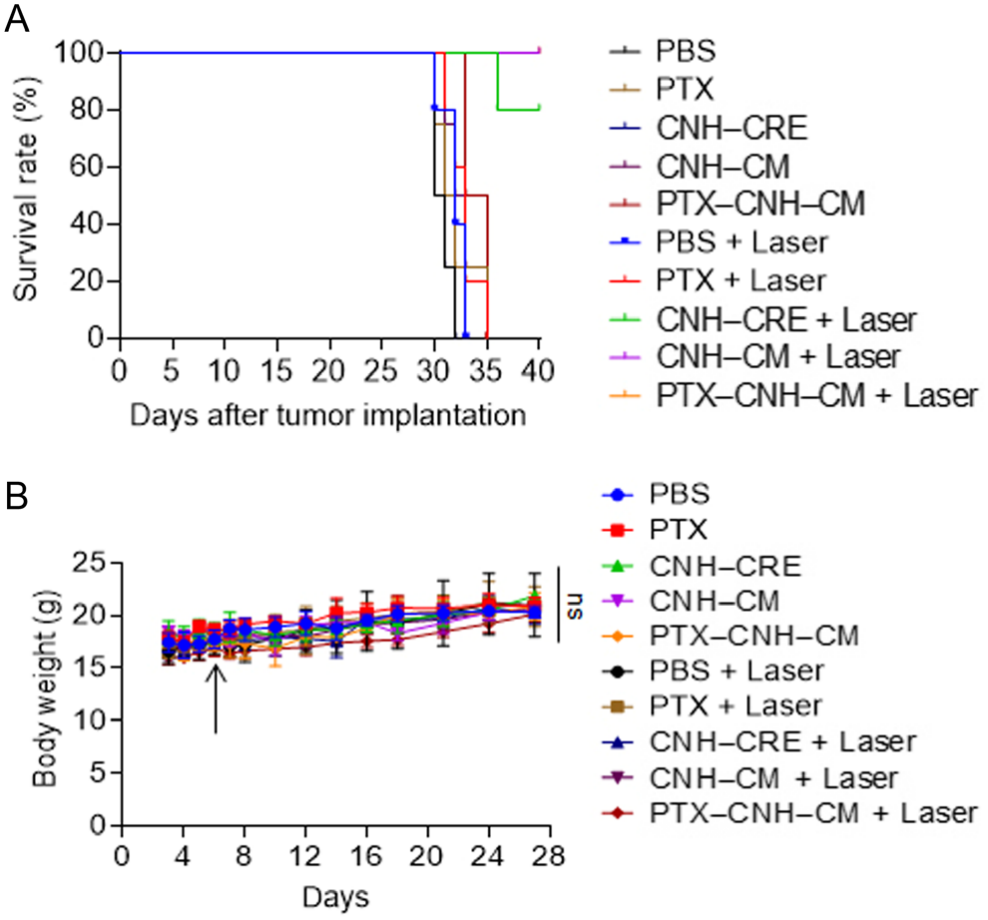
Survival rate and weight change in mice after treatment with light‐induced CNH complexes. A) Kaplan–Meier survival curves of Colon26‐tumor‐bearing mice (*n* ≥ 4 biologically independent mice) after tumor implantation for 40 days. Statistical significance was calculated in comparison with the PBS group. ***p < 0.001 by Log‐rank (Mantel–Cox) test. The PTX–CNH–CM and CNH–CM + laser groups showed a 100% survival rate for at least 30 days. B) Average mouse body weight after treatment during the treatment period. The black arrow displays the time point of sample administration.

Furthermore, the laser‐induced PTX–CNH–CM group exhibited not only excellent anticancer responses but also markedly prolonged survival rates (**Figure** **9**A). In contrast, the control groups without laser irradiation, CNH–CM and PTX, had somewhat anticancer effectiveness, even with 2‐shot administration, due to the immune response of the CM itself and the targeted delivery of PTX into the tumor by the EPR effect. However, PBS and CNH–CRE alone showed no therapeutic performance in prolonging the survival rate.

The body weight of all treatment groups, with and without laser, was relatively constant during the experimental period, indicating no side effects (Figure 9B). Furthermore, blood tests demonstrated that PTX–CNH–CM lacked in vivo toxicity (**Table** **1**). Significant differences were not observed in the complete blood count or biochemical parameters of the mice after intravenous injection with PBS or PTX–CNH–CM suspension after 30 days. These results indicate that laser‐induced PTX–CNH–CM was effective and safe as a multidimensional anticancer agent, and that both photothermal conversion and immunological stimulation could exert synergistic antitumor therapeutic effects for cancer treatment.

**Table 1:** Complete blood counts (CBCs) and biochemical parameters of the mice injected with PBS or PTX‐CNH‐CM dispersion after 7 days.

Measured value	Entry	Unit	PBS (*n* = 6)	PTX–CNH–CM (*n* = 7)	*P*‐value
CBC	WBC	×10^2^ μL^−1^	70.8 ± 17.92	73.14 ± 10.46	>0.05
	RBC	×10^4^ μL^−1^	869.8 ± 3.99	895.86 ± 31.16	>0.05
	HGB	g dL^−1^	13.68 ± 0.19	13.94 ± 0.3	>0.05
	HCT	%	40.06 ± 0.83	40.84 ± 1.27	>0.05
	MCV	fL	46.06 ± 0.49	45.6 ± 0.48	>0.05
	MCH	pg	15.74 ± 0.17	15.57 ± 0.35	>0.05
	MCHC	g dL^−1^	34.12 ± 0.27	34.16 ± 0.49	>0.05
	PLT	×10^4^ μL^−1^	84.54 ± 5.17	87.44 ± 9.47	>0.05
Biochemical parameters	TP	g dL^−1^	4.48 ± 0.13	4.4 ± 0.11	>0.05
	ALB	g dL^−1^	3.08 ± 0.08	3.16 ± 0.12	>0.05
	BUN	mg dL^−1^	24.02 ± 4.22	22.21 ± 1.49	>0.05
	CRE	mg dL^−1^	0.11 ± 0.01	0.11 ± 0.02	>0.05
	Na	mEq L^−1^	145.6 ± 0.89	145.75 ± 1.04	>0.05
	K	mEq L^−1^	20.44 ± 1.58	20.99 ± 0.57	>0.05
	Cl	mEq L^−1^	104 ± 1.22	105.25 ± 2.12	>0.05
	AST	IU L^−1^	41.6 ± 3.85	44.5 ± 4	>0.05
	ALT	IU L^−1^	20.4 ± 3.78	22.5 ± 2.98	>0.05
	LDH	IU L^−1^	184.6 ± 19.1	169.25 ± 19.57	>0.05
	AMY	IU L^−1^	2,112.4 ± 246.09	2029.5 ± 297.17	>0.05
	CK	IU L^−1^	71 ± 4.3	68.13 ± 25.59	>0.05

Data are represented as means ± SEM; *n* = 5 biologically independent mice. Statistical analyses comprise the two‐way ANOVA test.

ALB, albumin; ALT, alanine transaminase; AMY, amylase; AST, aspartate aminotransferase; BUN, blood urea nitrogen; Cl, chlorine; CK, creatine kinase; CRE, creatinine; HCT, hematocrit; HGB, hemoglobin; K, potassium; LDH, lactate dehydrogenase; MCH, mean corpuscular hemoglobin; MCHC, mean corpuscular hemoglobin concentration; MCV, mean corpuscular volume; Na, sodium; PLT, platelet; RBC, red blood cell; TP, total protein; WBC, white blood cell.

### 2.4 The Mechanism of Tumor Suppression by CNH Complexes

**Figure 10:**
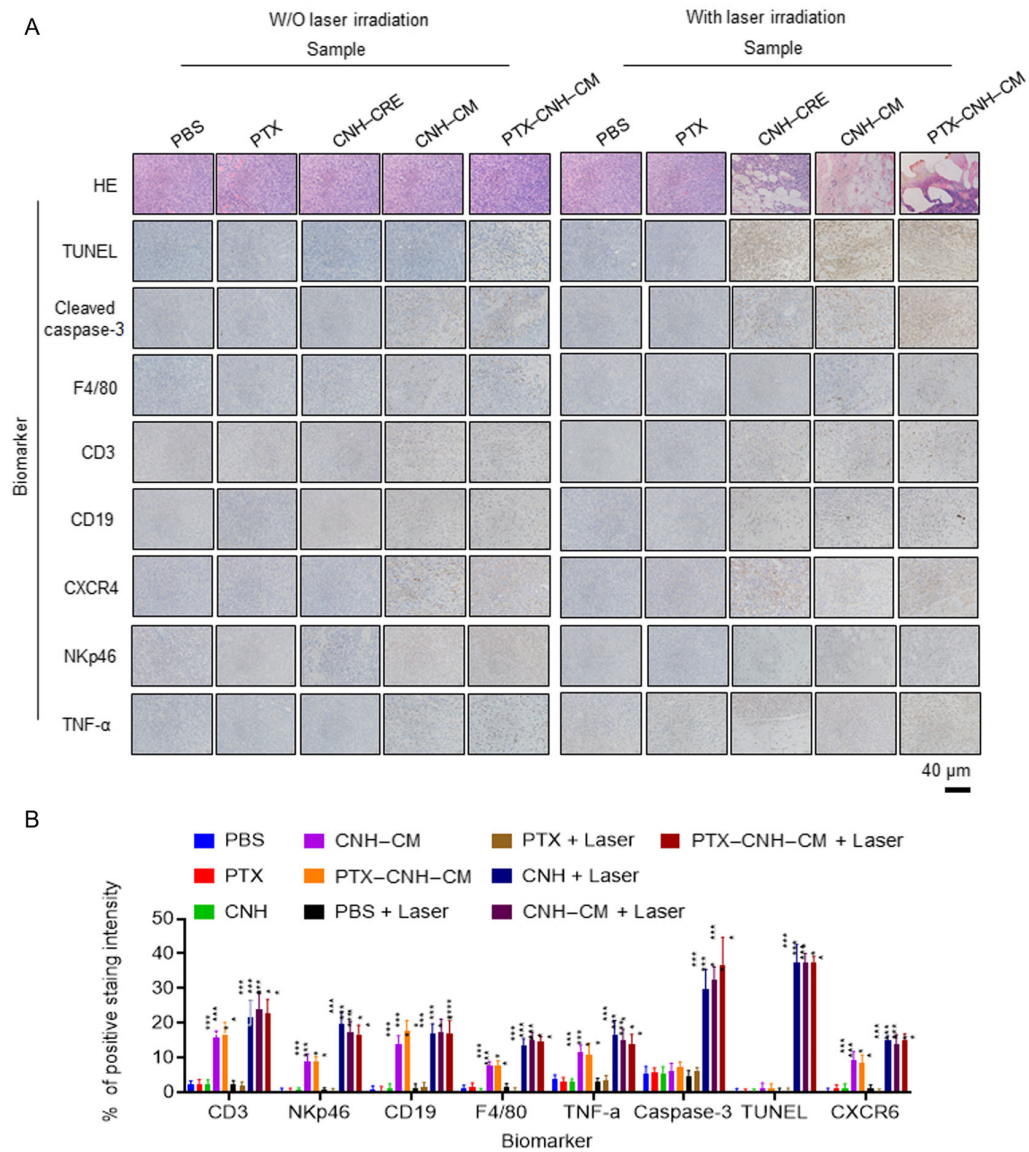
Mechanism of tumor suppression by laser‐driven CNH complexes. A) Hematoxylin and eosin (H&E), TUNEL, and IHC (caspase‐3, F4/80, CD3, CD19, CXCR4, NKp46, and TNF‐α) stained tumor tissues collected from different groups of mice on day 1 after their respective treatments. B) Intensity of color development in various IHC slides as a comparison of control and treated samples. Data are represented as mean ± standard error of the mean (SEM); *n* = 10 independent areas (region of interest) in each tumor tissue collected from the groups of mice 1 day after treatments. Statistical significance was calculated in comparison with the control group. **p < 0.01, ***p < 0.001, and ****p < 0.0001, by one‐way Student's *t*‐test.

To explore the immunological mechanism underlying the solid tumor regression induced by the light‐activatable CNH complex, hematoxylin and eosin (H&E) and immunohistochemical (IHC) staining analyses were performed (**Figure** **10**). In particular, the PTX–CNH–CM + laser group demonstrated obvious structural destruction of the solid tumor with intercellular fragmentation by the H&E staining assay, indicating strong antitumor efficacy. Meanwhile, the CNH–CRE + laser and CNH–CM + laser groups also showed tumor degradation, with anticancer therapeutic efficacy similar to that observed in the PTX–CNH–CM + laser group. In contrast, the H&E staining of tumor tissues in the PBS + laser and PTX + laser groups showed no discernible lesions throughout the treatment process. The terminal deoxynucleotidyl transferase‐mediated 2'‐deoxyuridine, 5'‐triphosphate nick end labeling (TUNEL) assay also confirmed the H&E staining results, revealing many apoptotic cells, especially in the PTX–CNH–CM + laser group. Simultaneously, the caspase‐3 staining assay further corroborated the strong anticancer mechanism of laser‐induced PTX–CNH–CM in vivo.

The immunological reactions were also observed in the CNH–CM and PTX–CNH–CM groups without laser (Figure 10), and the IHC staining of F4/80 (Macrophage marker), CD3 (T‐cell marker), CD19 (B‐cell marker), CXCR4 (neutrophil marker), NKp46 (NK cell marker), and TNF‐α results could support immune activation by the CMs. The other groups of PBS, PTX, and CNH–CRE were ineffective for immunological stimulation compared to PTX–CNH–CM and CNH–CM. IHC staining of the aforementioned immunological markers could also recognize the immunological reactions for modulating tumor regression by laser‐induced PTX–CNH–CM. Laser‐induced PTX–CNH–CM exhibited strong expression of all the immunological biomarkers, probably because both the laser and CM molecules could work together as stronger stimulants, especially for cytotoxic NK and T‐cells, respectively. Therefore, the CNH–CM and CNH–CM + laser groups also modulated NK and T‐cell markers due to the presence of CNH and the CM. The other control approaches of PTX + laser and PBS + laser were ineffective for immunological stimulation, as compared to PTX–CNH–CM + laser, CNH–CM + laser, and CNH–CRE + laser. Overall, the laser‐induced PTX–CNH–CM represents obvious tumor regression and fast healing of injury due to the synergistic NK and T‐cell stimulations and photothermal destruction of cancerous tumors by the effective apoptotic inhibition of PTX on cancer cells, the immune system activation by the CMs, and the excellent photothermal conversion and light‐activatable drug‐releasing properties of CNH. We believe that these synergistic immunological effects and optical nanofunctions of CNHs have a wide therapeutic application prospect.

## 3 Conclusion

In this study, we explored the use of CNHs as a functional material for developing a multimodal cancer phototheranostic platform. Although PTT alone can effectively ablate solid tumors under NIR irradiation, it has inherent limitations. Specifically, PTT may fail to eliminate cancer cells outside the irradiation region or metastatic diseases. To address these limitations, we developed cancer membrane‐wrapped CNH nanoparticles that can not only mediate targeted PTT but also act as immunotherapeutic agents. Building on this foundation, we further encapsulated the chemotherapeutic drug PTX within the CNH–CM complexes, creating a multimodal cancer phototheranostic platform that integrates PTT, immunotherapy, and chemotherapy. Our results demonstrated that the PTX‐loaded CNH–CM complexes achieved high accumulation and long retention at the tumor site. Furthermore, the nanocomplexes exhibited an enhanced PTX chemotherapeutic effect and a strong photothermal effect under laser irradiation. Notably, the immune analysis also showed that the CNH–CM carrier itself can activate various immune responses in the tumor. Therefore, our study shows that the biomimetic CNH complex system is an effective and comprehensive phototheranostic approach against malignant cancer. By combining the advantages of CNH with cancer CMs and the chemotherapeutic drug PTX in a three‐part system, we successfully achieved synergistic effects from the integration of PTT, immunotherapy, and chemotherapy. This multimodal platform can overcome the limitations of standalone PTT and provide a more effective and precise treatment for solid tumors and metastatic diseases.

## 4 Experimental Section

4.1

4.1.1

##### Nanocomplex Synthesis

The PTX–CNH–CM complexes were prepared by mixing 1 mg of CNH (average diameter, ≈80–100 nm; purity, 95%; metal‐free, NEC Corporation, Tokyo, Japan), 2 × 10^7^ Colon26 cells, and 1 mg of PTX in 10 mL of PBS, followed by pulse‐type sonication (VCX‐600; Sonics, Danbury, CT, USA) for 10 min in an ice bath. The CNH–CM nanoparticles were prepared similarly but without the addition of PTX. A PTX solution was separately prepared by dissolving 1 mg of PTX in 10 mL of PBS with 10% cremophor and sonicating for 5 min in an ice bath. Similarly, a CNH–CRE solution was prepared by dispersing 10 mg of CNH in 10 mL of PBS with 10% Cremophor EL (Nacalai Tesque, Kyoto, Japan) and sonicating for 10 min in an ice bath. Additionally, highly concentrated PTX–CNH–CM or PTX solutions were prepared by increasing the amounts of CNH and CM in the same ratio. The ICG–PTX–CNH–CM was prepared by mixing 1 mg of ICG (Tokyo Chemical Industry, Tokyo, Japan) with 1 mL of the prepared PTX–CNH–CM solution and vortexing for 1 min under dark conditions. The drug loading efficiencies of the PTX–CNH–CM and ICG–PTX–CNH–CM nanocomplex were assessed by calculating unbound PTX or ICG molecules after filtration using a UV–vis–NIR spectrometer. Briefly, the prepared aqueous suspension of PTX–CNH–CM or ICG–PTX–CNH–CM was filtered with a hydrophilic polyvinylidene fluoride filter membrane [pore size = 0.22 μm; Vented Millex‐GV Filter Unit (Sterile), Merck Millipore, Darmstadt, Germany]. The filtrates were measured using a UV–vis–NIR spectrometer to identify unbound PTX or ICG molecules and calculate drug loading efficiencies from calibration carves of PTX and ICG.

##### Structural and Optical Characterizations

The structural morphology and composition of the PTX–CNH–CM, CNH–CM, and CNH were meticulously examined employing a high‐resolution TEM (Model JEM‐2010; JEOL Ltd., Tokyo, Japan) operating at an acceleration voltage of 200 kV. These TEM investigations were conducted at the Hanaichi UltraStructure Research Institute Co., Ltd. in Aichi, Japan. DLS technology (Zetasizer Nano ZS; Malvern Panalytical, UK) was utilized to ascertain the hydrodynamic diameter and zeta potential of the nanoparticles. Additionally, the optical properties, specifically absorbance and FL, were characterized by deploying a UV–vis–NIR spectrophotometer (Model V‐730 BIO; Jasco Corporation, Tokyo, Japan) and an FL spectrometer (Model FP‐6300; Jasco Corporation, Tokyo, Japan), respectively. The amounts of CM and PTX conjugated with CNH were estimated by TGA (TG‐DTA8122; Rigaku, Tokyo, Japan). The PTX–CNH–CM and CNH–CM were prepared as the same way as the mentioned above but using 0.1 mg of PTX. TGA traces were recorded for the series using a ramp rate of 5 °C min^−1^ in air. The PTX–CNH–CM and CNH–CM were synthesized by the same way as the above protocol of nanocomplex synthesis except for using distilled water instead of PBS. The powder PTX–CNH–CM and CNH–CM was prepared by a freeze dryer (FDU‐1200; EYELA, Tokyo, Japan) before TG measurements.

Photothermal conversion efficacy: The photothermal transduction of PTX–CNH–CM nanoparticle dispersions was investigated by irradiating the solutions alongside MilliQ water with an 808 nm NIR laser (Civil Laser, Hangzhou, Zhejiang, China) at varying outputs of 1.2 W (≈61.1 mW mm^−2^, spot diameter ≈5 mm), 0.6 W (30.6 mW mm^−2^), and 0.3 W (15.3 mW mm^−2^). The thermal response of the solutions was continuously monitored in situ utilizing a high‐precision temperature sensor (Model AD‐5601 A; A&D Company, Tokyo, Japan). Furthermore, infrared thermographic imaging was conducted (Model i7; FLIR Systems, Nashua, NH, USA) to visually record the thermal patterns.

To evaluate the photothermal stability of the PTX–CNH–CM complexes, a 200 μL aliquot of the nanocomplex suspension was exposed to an 808 nm NIR laser at an intensity of 1.2 W (equivalent to ≈61.1 mW mm^−2^, with a spot diameter of around 5 mm) for 5 min. Subsequent to irradiation, the suspension was diluted with MilliQ water, and the resultant solution's optical absorbance spectrum was analyzed using a UV–vis–NIR spectrophotometer.

Photothermal conversion efficiency of nanocomplex was determined according to the previous methods.^[^ ^27^, ^34^, ^35^ ^]^ Detailed calculation was given as follows:
(1)

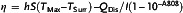

where *η* is the photothermal conversion efficiency of nanocomplex, *h* is the heat transfer coefficient, *S* is the surface area of the container, and the value of *hS* is obtained from the Equation (2). *T*_max_ is the maximum steady temperature of the solution of the nanocomplex and *T*_Surr_ is environmental temperature. *I* and *A*_808_ represent the laser power and the absorbance at the used laser wavelength (808 nm), respectively. *Q*_Dis_ expresses heat dissipated from the light absorbed by the solvent and container.

(2) $$hS=m_{D}C_{D}/\tau_{s}$$

where *m*_D_ and *C*_D_ are the mass of the solvent and the heat capacity of the solvent, respectively. A sample system time constant *τ*_s_ can be calculated by Equation (3).

(3) $$t\;=\;–\tau_{s}ln\left(\theta \right)$$

A dimensionless parameter *θ* is introduced as follows:
(4)

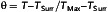




##### Controlled Drug Release

To assess controlled drug release, the PTX–CNH–CM complexes were subjected to an 808 nm NIR laser at an intensity of 1.2 W (≈61.1 mW mm^−2^, with a spot diameter of roughly 5 mm) for 20 min. Dispersion of the nanocomplexes was prepared in a 1 mL volume containing a concentration of CNH and PTX at 10 μg mL^−1^ each. The temporal release profile of PTX was monitored by collecting samples at specific intervals (0, 3, 5, 7, 10, and 20 min postirradiation). The samples were then filtered to procure the liberated PTX, which was quantitatively analyzed using a UV–vis–NIR spectrophotometer.

##### Cell Culture and Viability

Both the Colon26 and MRC5 cell lines were obtained from the Japanese Collection of Research Bioresources Cell Bank (Tokyo, Japan). The Colon26 cell line was cultured in Roswell Park Memorial Institute (RPMI) 1640 medium (Gibco, Grand Island, NY, USA) supplemented with 10% fetal bovine serum (FBS), 2 mM l‐glutamine, 1 mM sodium pyruvate, gentamycin, and 100 IU mL^−1^ penicillin–streptomycin. The MRC5 cells were cultured in Dulbecco's Modified Eagle's Medium (Gibco, Grand Island, NY, USA) supplemented with 10% FBS, 2 mM L‐glutamine, 1 mM sodium pyruvate, gentamycin, 100 IU mL^−1^ penicillin–streptomycin, and Hank's Balanced Salt Solution (Life Technologies, Carlsbad, CA, USA). Both cell lines were maintained at 37 °C in a humidified chamber containing 5% CO_2_. To prevent the genetic instability associated with high passage numbers, the cell stocks were regularly revived from cryopreserved vials stored in liquid nitrogen.

Cell viability was assessed using the Cell Counting Kit‐8 (CCK‐8) assay (Dojindo Laboratories, Kumamoto, Japan), following the manufacturer's instructions. Briefly, 1 × 10^4^ cells per well were seeded in 96‐well plates and allowed to adhere overnight. The cells were then exposed to the nanoparticles and irradiated with laser, as indicated. After washing with fresh medium, the cells were incubated with the CCK‐8 solution for 2 h at 37 °C. The absorbance at 450/690 nm was measured using a microplate reader.

##### Intracellular Penetration of the CNH Complex

Colon26 cells (1 × 10^5^ cells per well) were seeded in polylysine‐coated glass‐bottom dishes (Matsunami Glass, Osaka, Japan) and allowed to adhere overnight. The cells were then exposed to 10 μg mL^−1^ of ICG–PTX–CNH–CM (where the ICG, CNH, and PTX concentrations were 10, 10, and 1 μg mL^−1^, respectively) for 24 h at 37 °C in a humidified incubator containing 5% CO_2_.

The intracellular penetration of the ICG–PTX–CNH–CM was observed using an FL microscopy system (IX73) equipped with a mirror unit (IRDYE800‐33LP‐A‐U01; Semrock, Lake Forest, IL, USA) and an objective (40× magnification, 0.95 numerical aperture; UPLSAPO20X, Olympus) at room temperature. For 3D FL bioimaging, the Colon26 cells were incubated with ICG–PTX–CNH–CM in a similar manner. After three washes with PBS, the cells were examined, and images were acquired using a FL microscope (BZ‐X800, Keyence, Tokyo, Japan) and 3D analysis software (Keyence).

##### Cell Culture and Nanocomplex Treatment

Cells at a density of 1 × 10^5^ cells mL^−1^ were seeded into bioimaging dishes and incubated overnight to allow for adherence. A suspension of ICG–PTX–CNH–CM nanoparticles in PBS (with a CNH concentration of 10 μg mL^−1^) was prepared and introduced to the cells. Alternatively, an equivalent volume of PBS was added as a control. The cells were then incubated for 2 h at 37 °C in a 5% CO_2_ atmosphere. After three washes with PBS, the cells were maintained in RPMI medium.

A configured laser setup was employed to evaluate the destroyed cancer cells caused by laser‐activated nanomedicine. An 808 nm NIR continuous‐wave diode laser with an output of 254 mW (equating to an intensity of ≈129 mW mm^−2^) was integrated into an IX73 microscopy system (Olympus, Tokyo, Japan). A focused laser beam of 50 μm in diameter was directed onto the cells using a 40× objective lens with a numerical aperture of 0.95 (UPLSAPO40X, Olympus). The irradiation was conducted at room temperature for 5 s. Pre‐ and during irradiation images were captured with an electron‐multiplying charge‐coupled device camera (DP80, Olympus).

##### In Vivo Fluorescent Bioimaging

The animal experiments were conducted following protocols approved by the Institutional Animal Care and Use Committee of the Japan Advanced Institute of Science and Technology (JAIST) (No. 06‐001). To monitor the chronological changes in FL intensity due to the PTX–CNH–CM tumor‐targeting effect, Colon26 tumor‐bearing female BALB/cCrSlc mice (6 weeks old, *n* = 4, average weight = 18 g, average tumor size = 100 mm^3^) were intravenously injected with 200 μL of PBS or PBS containing ICG–PTX–CNH–CM (200 μL, ICG at 11.11 mg kg^−1^, PTX at 2.5 mg kg^−1^, and CNH at 10 mg kg^−1^, or 200 μL, ICG at 1 mg mL^−1^, PTX at 0.25 mg mL^−1^, and CNH at 1 mg kg^−1^). The mice were sacrificed, and the major organs (heart, liver, spleen, and kidneys) as well as the tumors were imaged using an in vivo FL imaging system (VISQUE InVivo Smart‐LF, Vieworks, Anyang, Republic of Korea) with a 3 s exposure time and an ICG filter (excitation: 740–790 nm, emission: 810–860 nm). The FL images were acquired and analyzed using CleVue software at 4, 8, 12, and 24 h postinjection (Vieworks).

##### In Vivo Anticancer Therapy

Male BALB/cCrSlc mice (*n* = 65, 6 weeks old, average weight = 15–20 g) were obtained from Japan SLC (Hamamatsu, Japan). Colon26 cell‐derived tumors were induced by subcutaneous injecting a 1:1 mixture of Matrigel (Dow Corning, Corning, NY, USA) and culture medium containing 1 × 10^7^ cells into the right dorsal side of the mice. After ≈1 week, when the tumor volume reached ≈100 mm^3^, the mice were intravenously injected with 200 μL of PBS, 200 μL of PBS containing PTX–CNH–CM (PTX at 0.25 mg mL^−1^ or 2.5 mg kg^−1^, CNH at 1 mg mL^−1^ or 10 mg kg^−1^), 200 μL of PBS containing CNH–CM (CNH at 1 mg mL^−1^ or 10 mg kg^−1^), 200 μL of PBS containing CNH (1 mg mL^−1^ or 10 mg kg^−1^), or 200 μL of PBS containing PTX (0.25 mg mL^−1^ or 2.5 mg kg^−1^). The dorsal right‐side tumors were irradiated for 5 min every other day, starting 24 h after sample injection, for a total of six laser irradiation sessions, using an 808 nm laser (600 mW, 30.6 mW mm^−2^).

Thermographic measurements were conducted during laser irradiation using infrared thermography. Tumor formation and overall health (viability and body weight) were monitored every other day. Tumor volumes were calculated using the formula *V* = *L* × *W* ^2^/2, where *L* and *W* represent tumor length and width, respectively. Mice were euthanized when the tumor volumes exceeded 1500 mm^3^, following the guidelines of the JAIST Institutional Animal Care and Use Committee.

IHC staining of tumor tissues: Colon26 tumor‐bearing mice (*n* = 5) were euthanized one day after the sample intravenous injection and laser irradiation. The tumor tissues from the treatment groups were then harvested for IHC staining. IHC analysis was performed by the Biopathology Institute Co., Ltd. (Oita, Japan) using standard protocols. Briefly, the primary tumors were surgically removed, fixed in 10% formalin, processed for paraffin embedding, and cut into 3–4 μm sections. After incubation with the primary antibody (see table in Supporting Information), the sections were stained with H&E and examined by light microscopy (IX73). The areas showing positive staining in the tumor tissues were analyzed using a light microscopy system (BZ‐X800) and hybrid cell count and microcell count software (Keyence). The color development of ten independent areas (regions of interest) was analyzed for each treated tumor tissue.

##### Statistical Analysis

All experiments were performed in triplicate and repeated three or more times. Quantitative values are shown as the mean ± standard deviation of at least three independent experiments. Statistical differences were performed using the Student's one‐sided/two‐sided *t*‐test. *P*‐values <0.05 were considered significant, and *P*‐values <0.01 were considered highly significant.

## Conflict of Interest

The authors declare no conflict of interest.

## Supporting information

Supplementary Material

## Data Availability

The data that support the findings of this study are available from the corresponding author upon reasonable request.
