# Supplementary material for: Biomimetic Functional Nanocomplexes for Photothermal Cancer Chemoimmunotheranostics
Source: Small Sci. 2024 Aug 19;4(10):2400324. doi: 10.1002/smsc.202400324 (PMC11935030; doi:10.1002/smsc.202400324)
Supplement: Supplementary file 1 — Supplementary Material [file SMSC-4-2400324-s001.zip › smsc.202400324-sup-0001-suppdata-S1.pdf]

WILEY-VCH

Supporting Information

Biomimetic functional nanocomplexes for photothermal cancer chemo-immunotheranostics

*Nina Sang, Yun Qi, Shun Nishimura, and Eijiro Miyako**

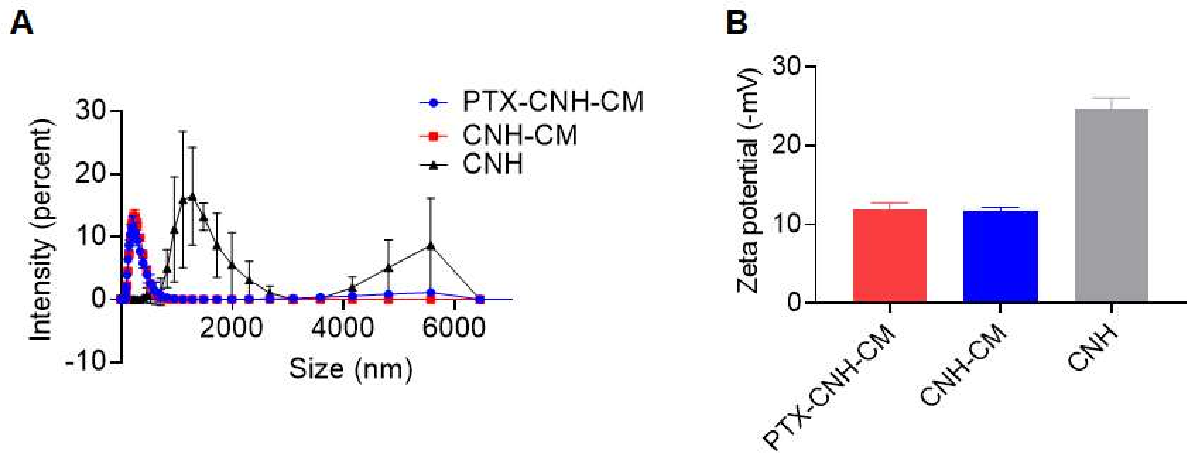

Figure S1. Characterization of functional CNH complexes. (A) Dynamic light scattering (DLS) (A) and zeta potential (B) measurements of PTX–CNH–CM, CNH–CM, and CNH.

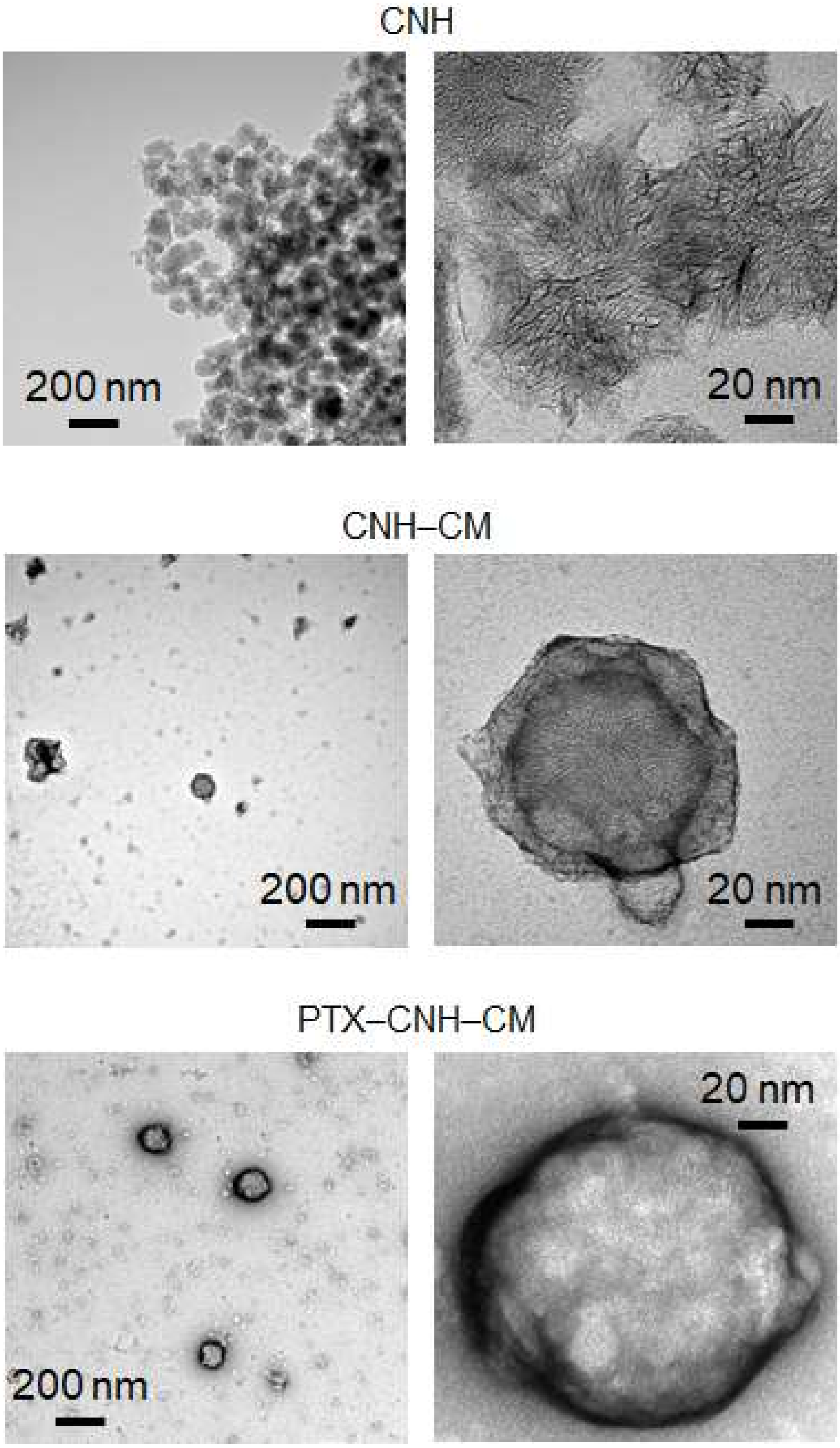

Figure S2. TEM observations of CNH, CNH–CM, and PTX–CNH–CM.

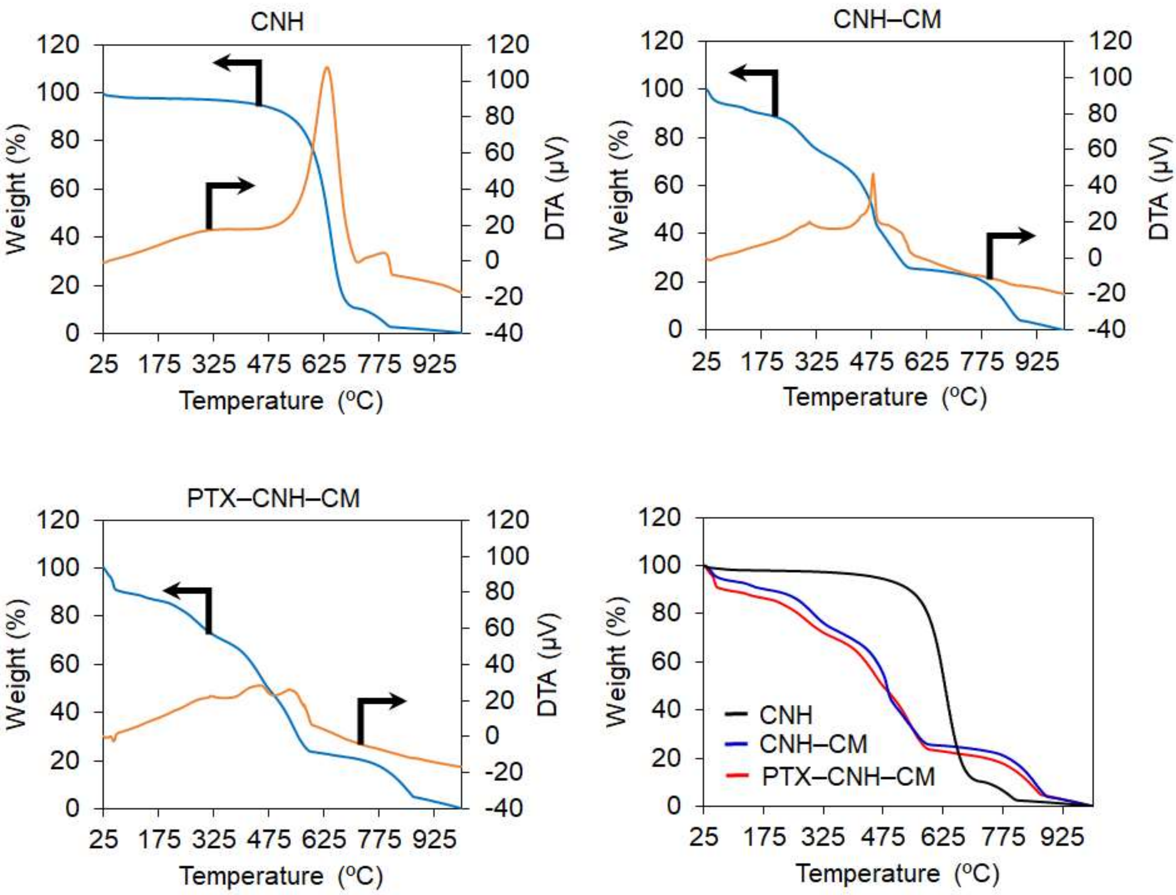

Figure S3. Thermogravimetric analysis (TGA) of CNH, CNH–CM, and PTX–CNH–CM.

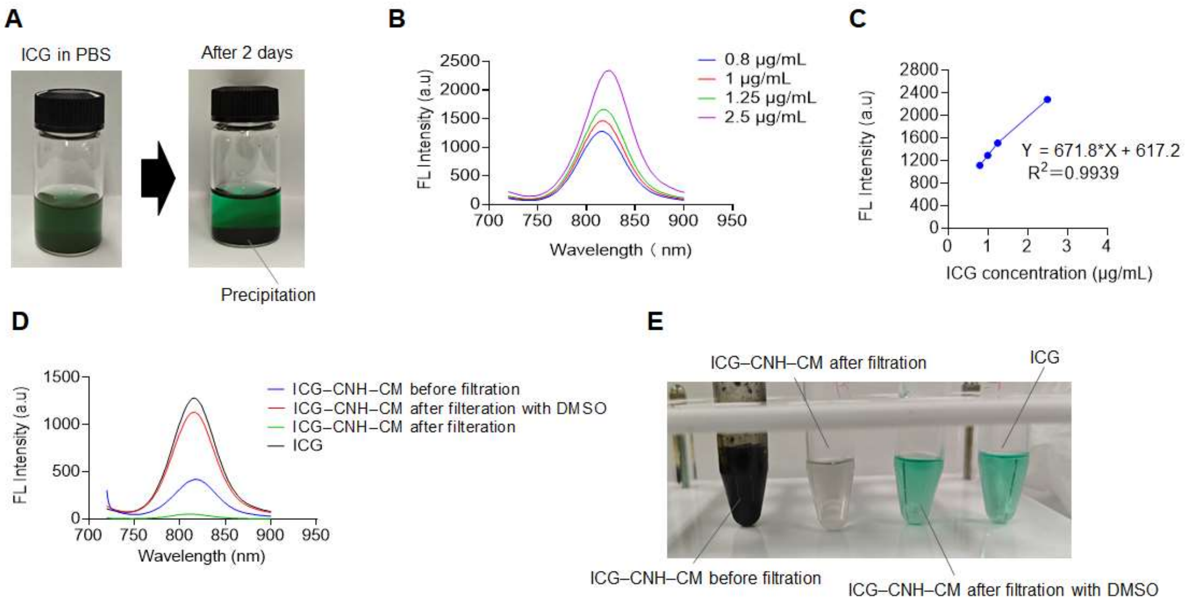

Figure S4. Loading efficiency of ICG into CNH–CM. (A) Photo of aqueous solutions of ICG in PBS just after preparation (left) and after 2 days at 20°C (right). ICG forms visible aggregations and massive precipitations in PBS. (B) Fluorescence (FL) intensity of ICG solution in DMSO at different concentration. (C) Calibration curve of ICG solution in DMSO. (D) FL intensity of each sample before and after treatments. Concentration of ICG was adjusted before treatment. (E) Photo of each sample before and after treatments.

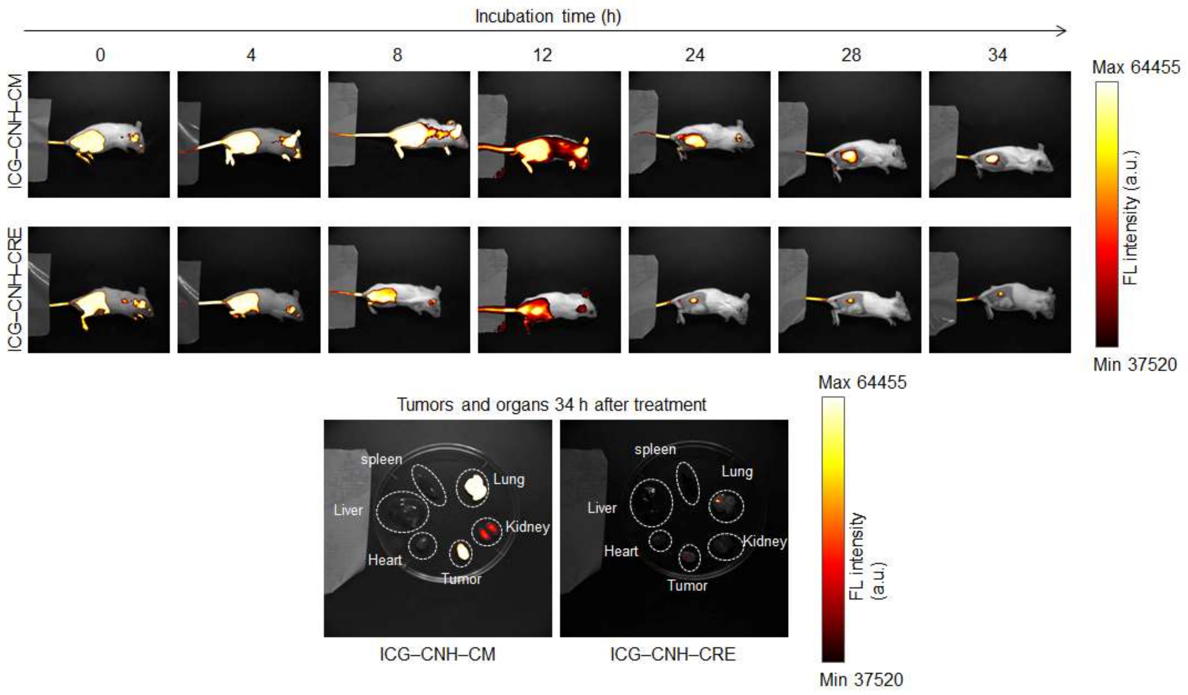

Figure S5. Systemic distribution of the CNH complex in the tumor model. A) FL imaging of Colon 26 tumor-bearing mice after i.v. injection of ICG–CNH–CM and ICG–CNH–CRE. Extracted vital organs and tumors after an i.v. injection of ICG–CNH–CM or ICG–CNH–CRE (ICG, 11.11 mg/kg; and CNH, 5.6 g/kg) (200 µL, ICG, 1 mg/mL; and CNH, 1 mg/mL).

Table S1. The photothermal conversion efficiency of materials in previous reports

Material	Photothermal conversion efficiency (%)	Reference
PTX–CNH–CM	63	This study
Gold nanorods	21	25
Gold nanoshells	13	25
Copper selenide	22	25
Carbon dots	31	26
Semiconducting polymer nanoparticles	37	27

Supporting Video legends

Supporting Video S1. Real time observation of cancer cell destruction by laser-induced CNH complex.

Supporting Video S2. Laser-induced cancer cells without CNH complex.
